# Effect of a Different Number of Amine-Functional Groups on the Gas Sorption and Permeation Behavior of a Hybrid Membrane Comprising of Impregnated Linde T and 4,4′- (Hexafluoroisopropylidene) Diphthalic Anhydride-Derived Polyimide

**DOI:** 10.3390/polym11111807

**Published:** 2019-11-04

**Authors:** Norwahyu Jusoh, Yin Fong Yeong, Serene Sow Mun Lock, Noorfidza Yub Harun, Mohd Hizami Mohd Yusoff

**Affiliations:** 1 Centre for Contaminant Control & Utilization (CenCoU), Chemical Engineering Department, Universiti Teknologi PETRONAS, 32610 Bandar Seri Iskandar, Malaysia; 2 CO_2_ Research Centre (CO_2_RES), Chemical Engineering Department, Universiti Teknologi PETRONAS, 32610 Bandar Seri Iskandar, Malaysia; yinfong.yeong@utp.edu.my (Y.F.Y.); serene.lock@monash.edu (S.S.M.L.); 3 Chemical Engineering Discipline, School of Engineering, Monash University Malaysia, Jalan Lagoon Selatan, 47500 Bandar Sunway, Malaysia; 4 Centre of Urbanization and Resources Sustainability (CUReS), Chemical Engineering Department, Universiti Teknologi PETRONAS, 32610 Bandar Seri Iskandar, Malaysia; noorfidza.yub@utp.edu.my; 5 Centre for Biofuel and Biochemical Research (CBBR), Chemical Engineering Department, Universiti Teknologi PETRONAS, 32610 Bandar Seri Iskandar, Malaysia; hizami.yusoff@utp.edu.my

**Keywords:** Impregnated Linde T, 6FDA-derived polyimide, hybrid membrane, sorption, CO_2_/CH_4_ separation

## Abstract

The bottleneck of conventional polymeric membranes applied in industry has a tradeoff between permeability and selectivity that deters its widespread expansion. This can be circumvented through a hybrid membrane that utilizes the advantages of inorganic and polymer materials to improve the gas separation performance. The approach can be further enhanced through the incorporation of amine-impregnated fillers that has the potential to minimize defects while simultaneously enhancing gas affinity. An innovative combination between impregnated Linde T with different numbers of amine-functional groups (i.e., monoamine, diamine, and triamine) and 4,4′-(hexafluoroisopropylidene) diphthalic anhydride (6FDA)-derived polyimide has been elucidated to explore its potential in CO_2_/CH_4_ separation. Detailed physical properties (i.e., free volume and glass transition temperature) and gas transport behavior (i.e., solubility, permeability, and diffusivity) of the fabricated membranes have been examined to unveil the effect of different numbers of amine-functional groups in Linde T fillers. It was found that a hybrid membrane impregnated with Linde T using a diamine functional group demonstrated the highest improvement compared to a pristine polyimide with 3.75- and 1.75-fold enhancements in CO_2_/CH_4_ selectivities and CO_2_ permeability, respectively, which successfully lies on the 2008 Robeson’s upper bound. The novel coupling of diamine-impregnated Linde T and 6FDA-derived polyimide is a promising candidate for application in large-scale CO_2_ removal processes.

## 1. Introduction

Natural gas has been identified as a primary fuel for the near future due to its low emissions and eco-friendly benefits. However, natural gas generally contains a considerable amount of CO_2_ impurities, which may create severe corrosion issue when associated with water, decrease the heating value of natural gas, reduce the pipeline capacity, and increase the greenhouse gas effect [1]. The future expansion of natural gas consumption and exhaustion of conventional resources will drive the exploration for low quality natural gas reserves. In the United States, it has been reported that 48 states contain oil fields with a high CO_2_ concentration [2]. The recovery of natural gas in those oil fields is forecast to gradually deplete and natural gas with a lower quality is predicted to be produced in the future. 

Membrane technology has become an alternative and attractive technique for removing CO_2_ from natural gas compared to existing technologies, such as absorption, adsorption, and cryogenic processes [3]. The first generation of membranes for CO_2_ separation was commercialized in 1983 through the utilization of a polymeric membrane [4]. Membrane technology requires a small space and is low weight; has low labor intensity, maintenance, and minimum utility requirements; and is easily adapted to existing equipment. So far, over 107 membrane systems have been installed around the world for natural gas sweetening processes [5]. Therefore, the ability of membranes in gas separation has attracted researchers to continue developing advanced membrane materials to achieve their designated separation, which benefits both environmental and energy-related processes.

In the past few decades, numerous membrane materials have been employed for gas separation applications. The employment of a polymer membrane has become a subject of intense research since it promotes low material and processing cost, good mechanical strength, and is easily produced. Nonetheless, polymer membranes are constrained in terms of achieving good permeability and selectivity simultaneously [6], whereby the tradeoff relationship has long been acknowledged by Robeson [7,8]. To circumvent this limitation of polymer membranes, inorganic membranes have been studied due to their extraordinarily high permeability and selectivity traits in comparison to polymeric materials. However, it is realized that inorganic membranes are relatively challenging to commercialize at an industrial scale due to its difficulty and high cost in fabrication [9]. Therefore, recent research works devoted to the fabrication of hybrid membranes has emerged to utilize advantages of different materials to improve gas the separation performance as an ultimate outcome [10]. In this context, the incorporation of inorganic fillers into the polymer membrane serves to increase the affinity toward certain gases while changing the structure’s free volume through alterations in the polymer chain packing [11]. Although hybrid membranes have displayed great improvements in enhancing the separation performance of its pristine material, as well as exhibiting practical processing procedures and fabrication costs, non-ideal morphologies that arise when coupling polymer and inorganic materials together remain a major issue to be overcome [12]. Non-ideal causes that often result in defects in hybrid membrane material include challenges in adhesion, interfacial contact, and the dispersion between the polymer and inorganic phases [13].

Therefore, various approaches have been reported in the published literature to overcome the defects between polymer and inorganic materials, which consequently improve the separation properties of hybrid membranes. The most commonly reported approaches include priming, heat treatment, chemical functionalization of inorganic particles, or the addition of interfacial agents to enhance the physical interaction with the polymer matrix [13,14,15]. An amine-functionalization agent is normally used to chemically modify the surface of particles by acting as an integral chain linker between the polymer matrix and particles [13]. Interactions between polymers and an amine-functionalization agent are dependent upon the agent‘s reactivity or compatibility toward the polymer [16]. 

Tien-Binh et al. [17] reported the CO_2_/CH_4_ gas permeability and selectivity of polymers grafted with varying functional groups and amine-functionalized, aluminium-based metal organic frameworks (MOFs) and concluded that the polymer–filler interaction in functionalized structure is the key component to ensuring a good separation performance. In another work by Guo et al. [18], they reported improved CO_2_ permeability compared to the pure polymer membrane and a slight CO_2_/CH_4_ selectivity enhancement through the utilization of an amine-functionalized, titanium-based MOF as the filler to fabricate polysulfone hybrid membranes. In addition, Ghalei et al. [19] reported great CO_2_/N_2_ and CO_2_/CH_4_ selectivity improvements with a minimal loss in overall gas permeability in high free volume polymer of intrinsic microporosity (PIM) through the addition of amine-functionalized, nanosized-zirconium-based MOF additives. Ge at al. [20] synthesized hybrid membranes consisting of amine-modified, copper-based MOFs into a Pebax-1657 polymer, which showed a substantial improvement in CO_2_/N_2_ and CO_2_/CH_4_ selectivities at the expense of a slight decrease in gas permeability. Acknowledging the incorporation of amine-functionalized MOF crystals as an effective strategy toward enhancing CO_2_ affinity, in recent work by Jia et al. [21], they proposed a novel material comprising of amine-functionalized MOFs@GO as a filler in a mixed matrix membrane, whereby the MOFs nanocrystals were grown in situ on the surface of graphene oxide (GO) nanosheets prior to dispersing them in a polyimide (PI) phase. Gas separation results revealed that both the permeability and selectivity of CO_2_/N_2_ were significantly improved compared to the pristine membrane. 

Based on a review of published literature pertaining to the elucidation of amine-functionalization agents for enhancing the gas separation performance of hybrid membranes, it was found that a majority of the recent study has been devoted to the employment of different types of MOFs, whereas the progress with amine-functionalized zeolites to synthesize hybrid membranes has received less attention. Nonetheless, there should be a considerable research effort devoted to revisiting this concept due to its excellent improvement in the CO_2_ absorption capability of amine-functionalized, zeolite-based particles (e.g., Zeolite 13X, Zeolite Y, and Zeolite ZSM-5) [22,23,24,25,26]. Among all zeolite-based materials, Linde T, an intergrowth of offretite and erionite, is a highly promising material for CO_2_/CH_4_ separation, whose function is attributed to its higher quadruple moment and polarizability toward CO_2_ that enables a high gas sorption capacity [27]. Nonetheless, the functionalization potential of Linde T has received less attention by other research groups to date. In addition, most of the studies devoted to the elucidation of zeolites functionalization have merely been confined to inorganic materials, while not being incorporated as fillers in the polymeric membrane matrix to study their gas separation performance. Another issue is that the majority of studies have been solely concerned with the improvement of the permeability and selectivity of the membranes [17,18,19,20,21]. In fact, the detailed studies on permeability, solubility, and diffusivity of gases through membranes, which have been commonly reported for polymer membranes [28,29,30,31,32] or available for some unimpregnated hybrid membranes [33,34,35,36,37], are important for unveiling the effect of amine-impregnated fillers toward different domains of gas transport properties.

Therefore, in the present study, transport properties including permeability, solubility, and diffusivity of CO_2_ and CH_4_ gases have been investigated for membranes incorporated with impregnated Linde T having different numbers of amine-functional groups (i.e., monoamine, diamine, and triamine) via gas permeation and sorption tests. This information is required to gain insights into the effect of incorporating impregnated Linde T particles toward changes in the gas transport properties of CO_2_ and CH_4_ through the 4,4′- (Hexafluoroisopropylidene) Diphthalic Anhydride (6FDA)-derived polyimide matrix. Moreover, elucidation on the effect of incorporating impregnated Linde T particles with different numbers of amine functional groups on the membrane matrix’s physicochemical properties has not been reported so far. To the best of our knowledge, this is the first paper that has reported the effect of amine-functionalized Linde T particles in a polyimide polymer phase, which can be ultimately used to synthesize a novel hybrid membrane material for CO_2_/ CH_4_ separation.

## 2. Materials and Methods 

### 2.1. Material

**Figure 1:**
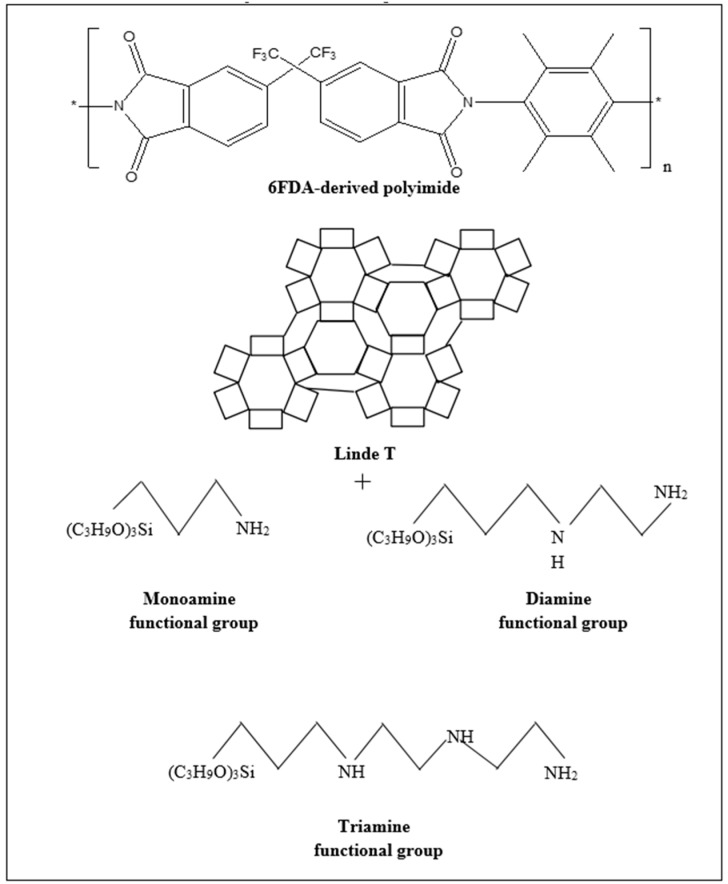
The chemical structures of 6FDA-derived polyimide, Linde T, and amine-functionalization groups, including monoamine (APTMS), diamine (AAPTMS), and triamine (AEPTMS) functional groups, adapted from Nafisi & Hägg (2014) and Huh et al. (2003) [40,41].

6FDA-derived polyimide and Linde T were synthesized from our laboratory via chemical imidization and ultrasonic-assisted hydrothermal growth methods, respectively, as reported in our previous publications [38,39]. 6FDA-derived polyimide consists of –C(CF_3_)_2_– and a bulky methyl group in its polymer backbone. These bulky functional groups contribute to a restriction of local segmental mobility and interchain packing, which results in the increase of separation performance. Preparation of impregnated Linde T required toluene (C_6_H_5_CH_3_, 99.8% purity, Sigma-Aldrich (M) Sdn. Bhd., Kuala Lumpur, Malaysia) and three different amine-functionalization groups including 3-(trimethoxysilyl)propylamine (APTMS, C_6_H_17_NO_3_Si, 97% purity, Sigma-Aldrich (M) Sdn. Bhd., Kuala Lumpur, Malaysia), N-[3-(trimethoxysilyl)propyl]ethylenediamine (AAPTMS, C_8_H_22_N_2_O_3_Si, >97% purity, Sigma-Aldrich (M) Sdn. Bhd., Kuala Lumpur, Malaysia) and N-[3-(trimethoxysilyl)propyl]diethylenetriamine (AEPTMS, C_10_H_27_N_3_O_3_Si, Sigma-Aldrich (M) Sdn. Bhd., Kuala Lumpur, Malaysia). All these chemicals were used as received. The chemical structures of the synthesized 6FDA-derived polyimide and Linde T, as well as three different amine functionalization groups, are shown in Figure 1 [40,41]. Regarding the fabrication of the membrane, dichloromethane (DCM, 99.8% purity, Sigma-Aldrich (M) Sdn. Bhd., Kuala Lumpur, Malaysia) was used without further purification. Moreover, purified carbon dioxide and methane gases with a 99.9995% purity were purchased from Air Products (M) Sdn. Bhd., Kuala Lumpur, Malaysia and utilized as received for the gas sorption and permeation experiments.

### 2.2. Preparation of Impregnated Linde T Crystals

Linde T particles were impregnated with monoamine, diamine, and triamine functional groups using APTMS, AAPTMS, and AEPTMS, respectively, by implementing the procedure reported by Li et al. [42]. First, 120 mL of toluene, 4.8 mL of amine-functionalization agent, and 0.6 g of particles were stirred for 24 h at room temperature under a nitrogen environment.

**Figure 2:**
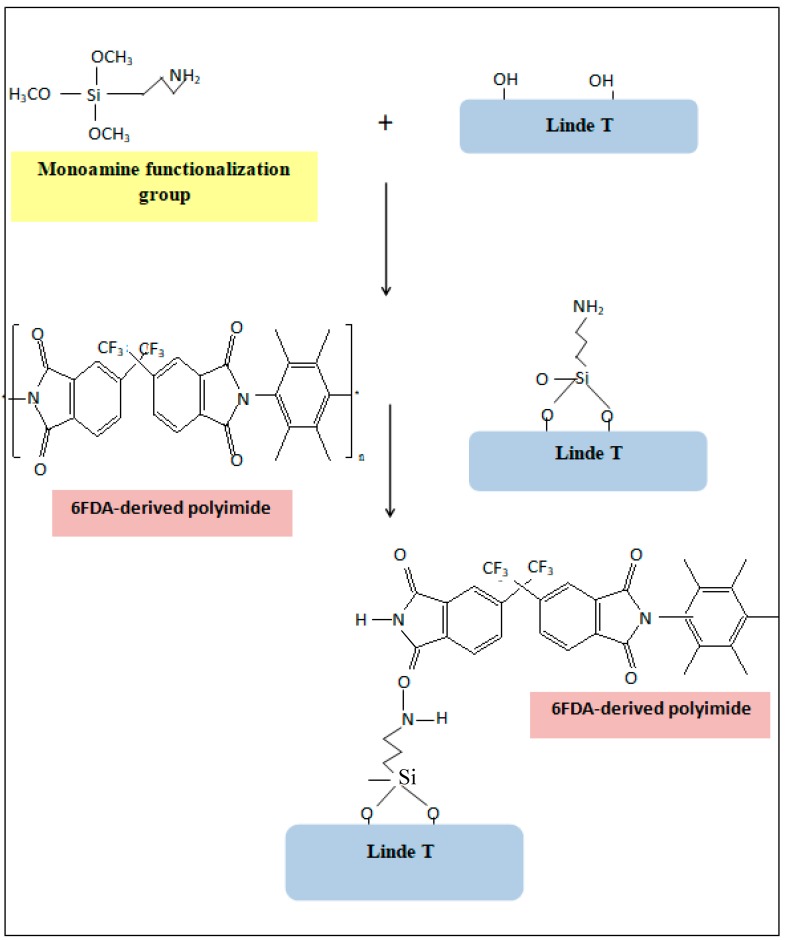
Amine functionalization agent reaction mechanism with 6FDA-derived polyimide and Linde T particles, adapted from Wahab et al. (2008) [43].

Particles were then recovered through centrifugation and washed with methanol to eradicate the unreacted silane after completion of the chemical modification process. The impregnated zeolite was dried at 110 °C for 24 h before use. The impregnated Linde T zeolite particles using APTMS, AAPTMS, and AEPTMS amine-functionalization agents were labeled as the 1A, 2A, and 3A particles, respectively. The amine functionalization group reaction mechanism between the Linde T and polymer phase is illustrated in Figure 2 [43].

### 2.3. Fabrication of Membranes

The pure 6FDA-derived polyimide membranes were fabricated by filtering 2% *w*/*v* of polymer solution prior to casting on a Petri dish. The polymeric membrane films were dried at ambient temperature overnight and further dried at 60 °C for 24 h under vacuum to ensure complete solvent evaporation. The films were then annealed at 250 °C for another 24 h under vacuum. Upon completion of the annealing process, the films were cooled slowly to room temperature under a vacuum condition and were stored in a moisture-free environment prior to use. 

On the other hand, hybrid membranes comprising of 1 wt% of impregnated Linde T particles and 6FDA-derived polyimide was fabricated via a dry-dry phase inversion method, as enclosed in our previous publication [44]. The polymer solution was added into the impregnated Linde T particle suspension prior to vigorous stirring for 1 h, followed by casting onto a Petri dish. The membrane film was peeled off and proceeded to the same heat treatment as applied for the pure 6FDA-derived polyimide membrane. The membranes embedded with impregnated Linde T particles using APTMS, AAPTMS, and AEPTMS were designated as the M1, M2, and M3 membranes, respectively.

### 2.4. Characterization of Membranes

A Zeiss Supra 55 VP field emission scanning electron microscope (FESEM) (Carl Zeiss NTS GmbH, Oberkochen, Germany) was used to investigate the morphology of the fabricated membranes. A cross-section of the membranes was obtained through freeze-fracturing of the samples after immersion in liquid nitrogen. A Quorum Q150R S sputter coater (Quasi-S Sdn. Bhd., Penang, Malaysia) was then used to coat the membrane film with platinum before analysis. On the other hand, filler distribution in the resultant membranes was obtained using an Oxford Instrument Inca energy (Oxford Instruments plc, Abingdon, UK) dispersion X-ray (EDX) through EDX mapping image and data analysis. In addition, an electronic balance supplied with a density measurement kit (Mettler Toledo, OHAUS CP224C, Shah Alam, Malaysia) was utilized to determine the density of the membrane via a buoyancy technique. A well-dried membrane sample was first weighted in air and an auxiliary liquid (high purity ethanol). From the measurements of weight in air and volume, the density of each polymer membrane was calculated. The membrane density was then used to calculate the fractional free volume (FFV) through the Bondi method using Equation (1) as follows [45]:

(1) $$FFV=\frac{V-1.3V_{w}}{V}$$

whereby *V* is the molar volume of the repeating unit of the polymer (cm^3^/mol) and *V*_w_ is the van der Walls volume (cm^3^/mol), which is calculated from the group contribution using the Bondi method.

A thermal analysis instrument based on differential scanning calorimetry (DSC) model Q2000 (TA Instruments, Eschborn, Germany) was utilized to measure the glass transition temperature (*T*_g_) of the membranes. During the first heating cycle, the samples were subjected to a heating rate of 10 °C·min^−1^ up to 450 °C in order to eliminate the thermal history of the membrane. Then, the membrane samples were quenched at a rate of 10 °C·min^−1^ before being reheated at 10 °C·min^−1^ in order to complete the second heating cycle. The *T*_g_ measurement was obtained from the second heating cycle.

### 2.5. Gas Sorption Tests

CO_2_ and CH_4_ solubilities were measured using a custom-built dual chamber sorption cell system based on a pressure decay methodology reported by Liu et al. [46]. The testing was conducted over a pressure range of 0–25 bar at a temperature of 30 °C. The ranges of pressure and temperature were set based on an operating condition commonly reported in literature for ease of comparison. Detailed elaboration of the sorption apparatus can be found elsewhere [47]. The system was comprised of two chambers, including reservoir and sorption cells, pressure and temperature transducers, a water bath, a heater, and a vacuum pump. 

The mass of the membrane sample was measured accurately and placed in the sample chamber before the entire system was vacuumed overnight to remove any gases and impurities trapped in the system. The sorption system was submerged in a water bath according to the desired experimental requirement where the temperature should be maintained and regulated between 25 °C and 50 °C. After completely evacuating both chambers, a test gas was then permitted to flow into the reservoir chamber at a desired pressure before it was introduced to the sorption cell. Pressures in the system were constantly examined via pressure transducers until equilibrium pressures were attained. Gas pressure was recorded automatically via a data acquisition system employing LabVIEW 2019 software (National Instruments, Kuala Lumpur, Malaysia). The amount of gas sorbed could be determined from the two equilibrium pressures using a gas law, as shown in Equation (2) [48]:

(2) $$n=\frac{PV}{ZRT}$$

where *n* refers to the amount of gas (mol), *P* is the pressure of the gas (MPa), *V* is the volume of the chamber (cm^3^), *Z* is the compressibility factor of the real gas under a certain designated temperature and pressure, *T* is the temperature of the gas (K), and *R* is molar gas constant (8.31446 cm^3^·MPa/K∙mol). 

Then, a stepwise pressure increase was applied for subsequent measurements at higher testing pressures and the entire cycle was reiterated to derive the whole sorption isotherm curve. The concentration of gas after solubility measurement could be obtained using Equation (3), as follows [49,50]:

(3) $$C=\frac{n\rho }{m_{P}}\times 22,414$$

where *C* represents the gas concentration (cm^3^(STP)/cm^3^ polymer), *ρ* is the polymer density (g/cm^3^), *m*_p_ refers to the mass of the polymer sample in the sorption chamber, and 22,414 cm^3^ (STP)/mole is a conversion factor.

The solubility of a membrane was determined using Equation (4), as follows [51]:

(4) $$S=\frac{C}{P}$$

where *S* is the solubility (cm^3^(STP)/cm^3^·bar) and *P* represents the pressure of the gas (bar). Then, the solubility selectivity, which represents the intrinsic capability of the membrane to exclude one gas from another based on the relative condensabilities of the two gases, was calculated using Equation (5), as follows:

(5) $$\alpha_{S,CO_{2}/CH_{4}}=\frac{C}{P}$$

where *α_s_* specifies the solubility selectivity of CO_2_/CH_4_. 

### 2.6. Gas Permeation Measurements

The permeation of CO_2_ and CH_4_ gases was measured under isothermal condition at 30 °C and a pressure of 3.5 bar using a custom-built gas permeation test rig under a dead-end flow system. Details of the experimental setup can be obtained elsewhere [52]. The permeation apparatus was comprised of a mass flow controller, pressure transducer, temperature controller, oven, back pressure regulator, vacuum pump, and bubble flow meter. 

A flat sheet membrane with an effective area of 1.77 cm^2^ was mounted onto the membrane test cell prior to subjecting the system under a vacuum condition overnight. The flow rate of gases was maintained at 200 mL/min using mass flow controllers. A test gas was introduced to the membrane cell, which was placed in an oven at a constant temperature. The outlet gas at the permeate stream was connected to a bubble flow meter for the flow rate measurement. The permeability of CH_4_ and CO_2_ gases were then obtained using Equation (6), as follows [53]:

(6) $$P_{A}=\frac{V_{P}t}{A_{m}\left(p_{f}-p_{p}\right)}$$

where *P*_A_*, V*_p_, *t*, and *A*_m_ correspond to the gas permeability (barrer), permeate flow rate at standard temperature and pressure (STP, 0 °C, 1 atm) (cm^3^(STP)/s), membrane thickness (cm), and membrane area (cm^2^), respectively. Meanwhile, *p*_f_ and *p*_p_ refer to the pressure at the feed and permeate side (cmHg), respectively. The subscript *A* is for CO_2_ or CH_4_. Membrane permeability is expressed in barrer unit (1 barrer = 1×10^−10^cm^3^(STP)·cm/s·cm^2^·cmHg).

The CO_2_/CH_4_ ideal selectivity, which is defined as the permeability ratio of CO_2_ and CH_4_ gases, was obtained using Equation (7), as follows [54]:

(7) $$\alpha_{{\mathrm{CO}}_{2}/{\mathrm{CH}}_{4}}=\frac{P_{{\mathrm{CO}}_{2}}}{P_{{\mathrm{CH}}_{4}}}$$

where α specifies the ideal selectivity of CO_2_/CH_4_ and *P* refers to the permeability (barrer). 

### 2.7. Gas Diffusivity Measurements

Based on gas transport mechanisms, the relationship between solubility, permeability, and diffusivity can be represented by Equation (8), as follows [51]:

(8) $$D=\frac{P}{S}$$

where *P*, *D*, and *S* indicate the permeability (cm^3^ (STP)·cm/s·cm^2^·cmHg), diffusivity (cm^2^/s), and solubility (cm^3^(STP)/cm^3^·cmHg), respectively. Therefore, the diffusivity of the membrane can be obtained using the value of solubility and permeability determined earlier in the previous sections. 

Then, the diffusivity selectivity was determined using Equation (9), as follows:

(9) $$\alpha_{D,{\mathrm{CO}}_{2}/{\mathrm{CH}}_{4}}=\frac{D_{{\mathrm{CO}}_{2}}}{D_{{\mathrm{CH}}_{4}}}$$

where α_D_ specifies the diffusivity selectivity of CO_2_/CH_4_.

## 3. Results and Discussion

### 3.1. Membrane Characterization

**Figure 3:**
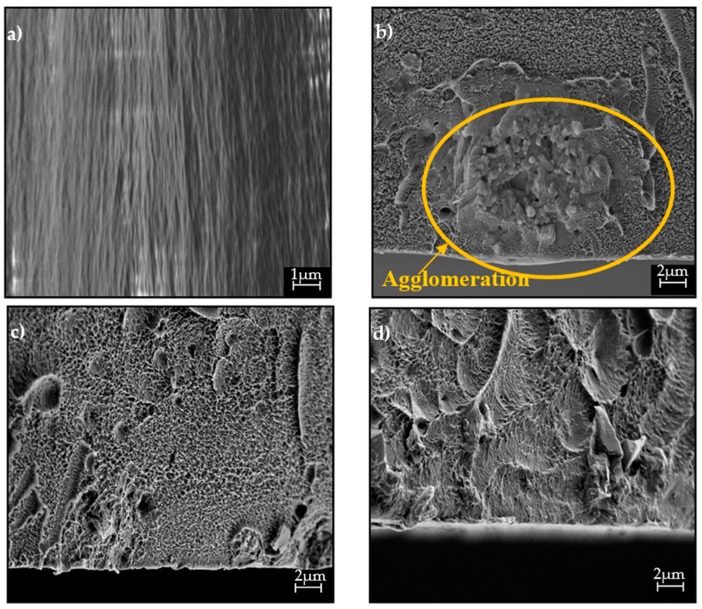
Cross-section images of (**a**) pure 6FDA-derived polyimide membrane, and impregnated Linde T (**b**) M1 (**c**) M2, and (**d**) M3 hybrid membranes.

**Figure 4:**
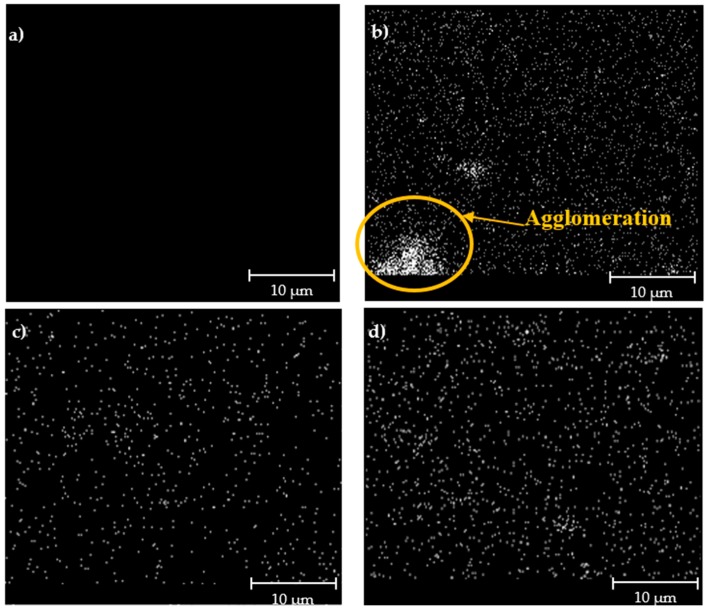
EDX-mapping images of (**a**) pure 6FDA-derived polyimide membrane, and impregnated Linde T (**b**) M1 (**c**) M2, and (**d**) M3 hybrid membranes.

The FESEM images of the hybrid membranes incorporated with impregnated Linde T particles are displayed in Figure 3. Based on Figure 3, it can be seen that the particles were sheathed in a fluorinated polyimide matrix for all the resultant membranes. Nevertheless, as the impregnated Linde T with a monoamine functional group (A1 particles) was amalgamated into the polymer matrix, particle sedimentation at the bottom of the membrane layer was observed. EDX-mapping of elemental Si in Figure 4b reconfirms a poor distribution of A1 particles in the polymer matrix for membranes embedded with A1 particles (M1 membrane). This result might have arisen from a weak interaction between the Linde T surface and monoamine functional group, which consequently increased the challenges for uniform particle dispersion within the polymer matrix [55].

On the contrary, particle sedimentation, as well as the formation of pinholes and voids, was not found when the impregnated Linde T with diamine (A2 particles) and triamine (A3 particles) functional groups were amalgamated into the polymer phase. This observation reflected the improvement of polymer–filler interfacial adhesion by incorporating A2 and A3 particles into the polymer matrix compared to A1 particles. In addition, the incorporation of A1 and A2 particles eradicated the filler–polymer interfacial voids and gaps, which ameliorated the distribution of particles in the polymer phase while inhibiting sedimentation. A good distribution of impregnated Linde T particles in the M2 and M3 membranes were further verified through EDX mapping, as displayed in Figure 4c,d, respectively. This phenomenon was possibly due to the formation of stable amino functionalization-derived layers on the surface of Linde T by diamine and triamine functional groups, which contributed to the improvement of particle dispersion in the polymer phase [56].

Table 1 demonstrates the densities and FFVs of the membranes fabricated in the present work. Reproducibility of the density and FFV values acquired were within the range of about ±0.01 g/cm^3^ and ±0.01, respectively. According to Table 1, the density and FFV values of the resultant membranes ranged from 1.27–1.29 g/cm^3^ and from 0.24–0.26, respectively, where the M2 membrane exhibited the highest FFV with 0.260. All the hybrid membranes displayed a lower density and higher FFV in comparison to the pure 6FDA-derived polyimide membrane. This behavior might be attributed to the increase of accessible cavities in the polymer phase through the inclusion of impregnated Linde T particles into the polymer matrix, which further increased the FFV. 

In addition, it can be found that the incorporation of A1 particles in the 6FDA-derived polyimide phase demonstrated the lowest FFV of 0.244 in comparison to the membranes loaded with A2 and A3 particles. This result shows that the impregnated Linde T surface with a monoamine functional group promoted a higher packing density in the polymer matrix, which resulted in the reduction of the void space as a whole. Nonetheless, M2 and M3 membranes displayed an enhancement in the FFV of up to 0.248 and 0.260, respectively when impregnated Linde T particles using diamine and triamine functional groups were embedded in the membrane compared to the M1 membrane. The FFV enhancement might be attributed to the efficient chain packing alteration by A2 and A3 particles, which subsequently improved the free volume and accessible cavities in the polymer matrix.

DSC results of the resultant membranes are shown in Table 1. The experimental errors of the *T*_g_ values obtained from DSC measurements was about ±0.1 °C. Based on Table 1, all the hybrid membranes showed a higher *T*_g_ value of up to 426 °C compared to the pure 6FDA-derived polyimide with a *T*_g_ of only 410 °C. This result indicates that the stiffness and rigidity of the polymer chain structure increased with the presence of impregnated Linde T particles in the membrane. The membranes incorporated with impregnated Linde T particles using diamine and triamine functional groups demonstrated higher *T*_g_s compared to the membrane loaded with impregnated Linde T particles using a monoamine functional group. The enhancement of *T*_g_ might be attributed to the increase of the polymer matrix rigidity when the membrane was loaded with impregnated Linde T particles with a higher number of amine functional group due to the enhancement of covalent interactions between polyimide chains and amine functional groups [55]. 

### 3.2. Determination of Gas Transport Properties of the Membrane

#### 3.2.1. Gas Sorption

**Figure 5:**
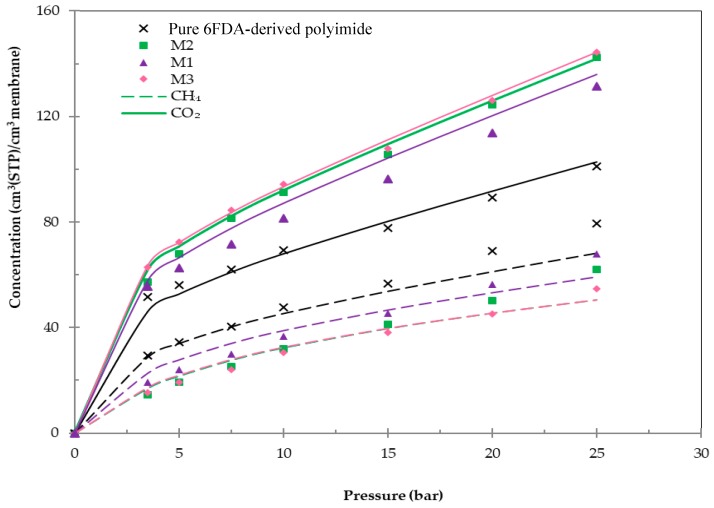
Sorption isotherms of pure 6FDA-derived polyimide and impregnated Linde T hybrid membranes including M1, M2, and M3 membranes at 30 °C.

Sorption isotherms of CO_2_ and CH_4_ for the fabricated membranes at a temperature of 30 °C are demonstrated in Figure 5. 

Referring to Figure 5, both CO_2_ and CH_4_ isotherms for all membranes demonstrated dual-sorption behavior, whereby the uptake rapidly increased in gas concentration at low pressure and tapered off at higher pressure. This behavior signified that the microvoid space within the membrane matrix was swiftly filled at low pressure [57]. However, as the pressure increased, free microvoid space became limited and the sorption of gas was only able to occur within the polymer matrix. In addition, it can be observed that the CO_2_ sorption for all membranes was higher compared to the CH_4_ sorption, which was in accordance with the increase of condensabilities of these gases. Furthermore, the higher critical temperature of CO_2_ and persuasive interaction between CO_2_ molecules and the membrane in comparison to CH_4_ might have also contributed to the enhancement of CO_2_ sorption in the membrane matrix [58].

Furthermore, the membranes embedded with impregnated Linde T particles demonstrated a greater CO_2_ sorption in comparison to the pure 6FDA-derived polyimide membrane, which signified that the capacity of CO_2_ sorption in the membranes was enhanced after loading with impregnated Linde T. This behavior can be associated with the increase of FFV and strong CO_2_-membrane contact in hybrid membranes compared to the pure 6FDA-derived polyimide membrane [59]. In addition, impregnation of Linde T introduced amine functional groups to the surface of inorganic particles, which may interact with more condensable gases, such as CO_2_, and thus increase the gas solubility in the membrane. On the other hand, CH_4_ sorption reduced with the amalgamation of impregnated Linde T into the polymer phase, which indicated a weak interaction between the membrane and CH_4_ molecules. 

The gas sorption of the resultant membranes at a pressure of 3.5 bars and temperature of 30 °C are displayed in Table 2. The experimental error of the attained solubility values was about ±0.5×10^−2^ cm^3^(STP)/cm^3^·cmHg.

Based on Table 2, CO_2_ and CH_4_ solubilities were obtained within the ranges (19.71–23.90) × 10^−2^ cm^3^(STP)/cm^3^·cmHg and (5.48–11.20) × 10^−2^ cm^3^(STP)/cm^3^·cmHg, respectively, for the fabricated membranes. CO_2_/CH_4_ solubility selectivity ranging from 1.76 to 3.98 was also attained for these membranes, whereby the M2 membrane demonstrated the highest CO_2_/CH_4_ solubility selectivity. Furthermore, it was observed that the sorption of CO_2_ followed the trend of M3 > M2 > M1 for hybrid membranes. The membrane incorporated with A1 particles demonstrated the lowest CO_2_ sorption, which might be explained through a partial blockage of the offretite structure opening and hindrance by the polymer chain [60]. This finding was consistent with the FFV value shown in Table 1, whereby the M1 membrane demonstrated the lowest FFV value compared to that of the M2 and M3 membranes. In fact, this behavior was reasonable because the monoamine functional group could coordinate to the silicon atom and form a stable five-membered cyclic intermediate for intramolecular catalysis, which subsequently lead to the creation of the least hydrolytically stable amine-functionalization layer [56]. Hence, the interaction of the polymer phase with an amine functional group was reduced. 

**Figure 6:**
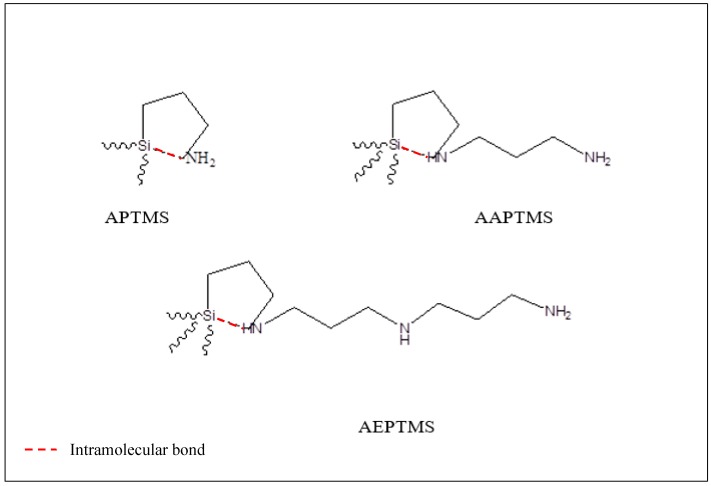
Intramolecular catalysis of amine-functionalization agents including APTMS (monomine), AAPTMS (diamine), and AEPTMS (triamine), adapted from Zhu et al. (2012) [56].

Figure 6 shows the ability of the different amine-functionalization groups for intramolecular catalysis [56]. Intramolecular catalysis is vital for silanization and hydrolysis, which can be described through the ability of the amine functionalization groups to catalyze siloxane bond hydrolysis through formation of five-membered cyclic intermediate. 

Referring to Figure 6, it can be observed that the amine functionality in the triamine functionalization group was less likely to be involved in intra-molecularly catalyzed siloxane bond hydrolysis because of the longer alkyl linker that separated the amine group and silicon atom compared to diamine and monoamine functionalization groups. Thus, membranes loaded with diamine and triamine functionalized particles resulted in the increase of CO_2_ sorption compared to the M1 membrane. The increase of CO_2_ sorption in M2 and M3 membranes was also in agreement with the results from the FFV value whereby M2 and M3 membranes exhibited higher FFV values than that of the M1 membrane. This phenomenon might be attributed to the capability of diamine and triamine groups to sterically hinder the bond detachment of intramolecular catalysis, which consequently led to the formation of a stable amine-functionalized surface, as demonstrated in Figure 6 [56]. In addition, it was found that the hybrid membrane embedded with A3 particles exhibited slightly higher CO_2_ and CH_4_ sorptions than those values obtained for the M2 membrane, which might be ascribed to the higher affinity and interaction between CO_2_/CH_4_ penetrants and the M3 membrane.

#### 3.2.2. Gas Permeation

Gas permeations of the fabricated membranes are tabulated in Table 2. Experimental error of the permeability values obtained was about ±2 barrer. According to Table 2, CO_2_ and CH_4_ permeabilities ranging from 765.3 barrer to 857.7 barrer and 31.1 barrer to 56.8 barrer, respectively, were attained for the resultant membranes. In addition, the CO_2_/CH_4_ selectivity ranging from 13.5 to 26.4 was also obtained for these membranes. The hybrid membrane embedded with impregnated Linde T zeolite using diamine functionalization agent demonstrated the highest CO_2_ permeability and CO_2_/CH_4_ selectivity of up to 821.0 barrer and 26.4, respectively. 

However, as A1 particles were loaded into the membrane, CO_2_ permeability and CO_2_/CH_4_ selectivity reduced by 9.3% and 29.5%, respectively, in comparison to the hybrid membrane incorporated with A2 particles. These results were in the agreement with the EDX-mapping images shown in Figure 4, whereby a poor filler distribution can be seen with the trivial agglomeration and sedimentation of Linde T particles at the bottom of the membrane layer. Moreover, the drop in *T*_g_ and FFV values of the M1 membrane also contributed to the reduction in gas permeability and CO_2_/CH_4_ selectivity. 

On the other hand, the membrane embedded with A2 particles had an increased CO_2_ permeability and CO_2_/CH_4_ selectivity of up to 821.0 barrer and 26.4, respectively. Furthermore, the incorporation of impregnated Linde T particles using a triamine functional group into a 6FDA-derived polyimide matrix also contributed to the enhancement of gas separation properties with a CO_2_ permeability of 858.0 barrer and a CO_2_/CH_4_ selectivity of 22.5. The increase of the gas separation performance for M2 and M3 membranes were also in agreement with the FESEM images and EDX-mapping shown in Figure 3 and Figure 4, respectively, in which a good distribution of Linde T particles in the polymer matrix was observed. In addition, as shown in Table 1, the M2 and M3 membranes showed an enhancement in *T*_g_, whereby the M2 membrane exhibited the highest increase in *T*_g_ and FFV values. The enhancement of *T*_g_ demonstrated the reduction of segmental chain mobility, which caused a lower penetration of the larger molecule (CH_4_) compared to the smaller molecule (CO_2_) through the membrane. Therefore, the CO_2_ permeability and CO_2_/CH_4_ selectivity increased. In addition, the *T*_g_ could be correlated to the FFV, in which the increase in microvoid formation led to a lower activation energy for permeability [61]. The increase of permeability with the enhancement of FFV is in agreement with results obtained by reported literature [62,63]. The improvement of permeability as a function of FFV can be described using Equation (10) [62]:

(10) $$P=A_{D}Se^{-B/FFV}=A_{P}e^{-B/FFV}$$

where *P* is the permeability, *A*_D_ is the constant with respect to the size and kinetic velocity of the penetrant, *B* refers to the free volume of holes needed for penetrant diffusion, *S* designates the solubility, and *A*_p_ is the product combination of *A*_D_ and *S*. Nevertheless, the performance of the M3 membrane was slightly lower than the M2 membrane, which might be contributed to the longer chain length of triamine compared to the diamine functional group.

#### 3.2.3. Gas Diffusion

After sorptions and permeabilities of the membranes were investigated, as explained in the previous section, diffusivity of the membrane was obtained using the value of solubility and permeability as represented in Equation (8). Table 2 displays the diffusivity, *D*, of CO_2_ and CH_4_ in pure 6FDA-derived polyimide, M1, M2, and M3 membranes at a pressure of 3.5 bars and temperature of 30 °C. Reproducibility of the attained diffusivity values was about ±0.5 × 10^−8^ cm^2^/s. According to Table 2, the CO_2_ and CH_4_ diffusivities ranged from (23.75–37.56) × 10^−8^ cm^2^/s and (5.94–7.72) × 10^−8^ cm^2^/s were obtained for the fabricated membranes. CO_2_/CH_4_ solubility selectivity ranging from 4.00 to 6.60 was also attained for these membranes, whereby the M2 membrane demonstrated the highest CO_2_/CH_4_ diffusivity selectivity.

In addition, in all cases, the diffusivity of CH_4_ was lower compared to CO_2_, which could be associated with the larger kinetic diameter of CH_4_ (3.8 Å) compared to CO_2_ (3.3 Å). Furthermore, the solubility, permeability, and diffusivity of all hybrid membranes were higher than those of the pure 6FDA-derived polyimide membrane, which was consistent with the increase in the FFV value for the membrane embedded with impregnated Linde T, as presented in the previous section. The increase of diffusivity with the enhancement of FFV is in agreement with the results obtained by Escorihuela et al. [64], Recio et al. [62], and Wang et al. [65]. On the other hand, based on Adams et al. [66] and Sadeghi et al. [67], the presence of particles in the polymer phase generally contributes to physical barriers in the membrane, whereby they act as a hindrance in the diffusive path of a gas molecule to penetrate through the membrane. These obstacles increase tortuosity for the gas molecules in diffusing through the membrane. Nevertheless, the distribution of particles inside the polymer phase also plays a role in the separation characteristics, in which well distributed particles create an accessible path for gas molecules and subsequently increases the diffusivity [64]. Based on EDX-mapping images shown in Figure 4, M2 and M3 membranes demonstrated good particle distributions, which supported the increase of gas diffusivity in the membrane. In contrast, the presence of particle agglomeration in the M1 membrane led to a limited channel accessibility for gas molecules, where the particles acted as barrier and subsequently resulted in a decrease of diffusivity. However, the increase of free volume in the M1 membrane compared to the pure fluorinated polyimide improved the diffusion process, which superseded the tortuosity effect induced by the particle addition.

Overall, despite the M2 membrane displaying a slight reduction in solubility and permeability of CO_2_ and CH_4_ compared to the M3 membrane, the incorporation of A2 particles in the polymer phase permitted higher selectivities of permeability, solubility, and diffusivity. It can be concluded that the improvement in selectivities of the hybrid membrane loaded with A2 particles might have been caused by the ability of aminosilane-functionalized Linde T zeolite particles to reduce the transportation of gases via the voids and subsequently enhance the selectivity of the membrane [68]. On the other hand, the M1 membrane demonstrated the lowest selectivities in permeability, solubility, and diffusivity. This result signifies that incorporation of A1 particles in the 6FDA-derived polyimide matrix did not successfully reduce the voids and thereby produced a decrease in the selectivity of the membrane. The reduction in performance of the M1 membrane was supported through FESEM images, EDX mapping, *T*_g_, and FFV results obtained in Figure 3 and Figure 4, and Table 1.

On the other hand, it can be observed that the gas solubility was much lower than the diffusivity. Thus, enhancement in CO_2_ permeability in hybrid membranes was attributed to the simultaneous improvement in solubility and diffusivity, whereby the overall permeability was caused by the increase in the diffusivity. 

### 3.3. Comparison of Membrane Performance with the Robeson Upper Bound 

**Figure 7:**
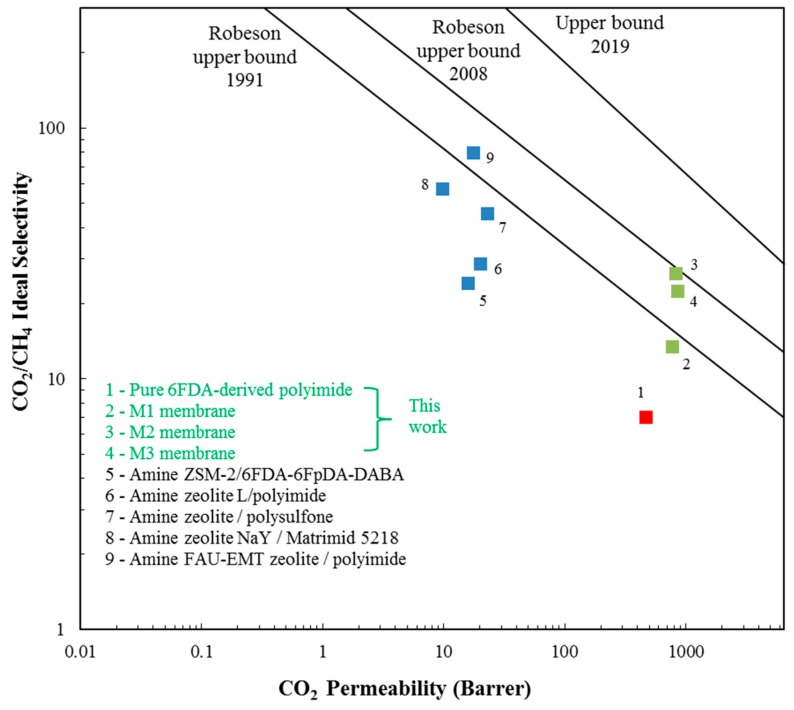
Gas separation performance of membranes fabricated in the present work compared with Robeson’s upper bound.

The separation performance of impregnated Linde T hybrid membranes fabricated in the present work was compared with the Robeson upper bound limit and the results are shown in Figure 7. 

As illustrated in Figure 7, it can be seen that the performance of the pure 6FDA-derived polyimide membrane obtained in this work lies below the Robeson upper bound limit from 1991. Although the integration of impregnated Linde T with a monoamine group in a 6FDA-derived polyimide phase (M1 membrane) successfully enhanced both the CO_2_ permeability and CO_2_/CH_4_ selectivity compared to the pure 6FDA-derived polyimide membrane, it was still unable to transcend the Robeson upper bound line from 1991. Nevertheless, as impregnated Linde T with a triamine functional group was embedded into the polymer matrix (M3 membrane), the membrane successfully surpassed the prior limit and approached the 2008 Robeson trade-off line. Meanwhile, the hybrid membrane incorporated with impregnated Linde T with a diamine (M2 membrane) successfully lies on the Robeson upper limit from 2008. However, the fabricated hybrid membranes were still unable to surpass the revised upper bound from 2019 [69].

Furthermore, Figure 7 compares the results of the CO_2_ permeability and CO_2_/CH_4_ selectivity of impregnated Linde T/6FDA-derived polyimide hybrid membranes with other published results [55,70,71,72,73] using various types of impregnated zeolite-based hybrid membranes. Based on Figure 6, hybrid membranes fabricated in the present work exhibited a higher performance than the impregnated zeolite-based hybrid membranes reported in the literature. This finding shows that impregnated Linde T/6FDA-derived polyimide membranes are potential candidates in separating CO_2_ from CH_4_.

## 4. Conclusions

In the present work, Linde T impregnated with different numbers of amine functional groups were embedded into a 6FDA-derived polyimide for CO_2_/CH_4_ separation. The transport properties, including solubility, permeability, and diffusivity, of the fabricated membranes were examined via gas sorption and permeation tests. The hybrid membrane incorporated with impregnated Linde T using a diamine functional group (M2 membrane) demonstrated the highest CO_2_/CH_4_ selectivities with solubility, permeability, and diffusivity of up to 26.35, 3.98, and 6.61 compared to membranes embedded with impregnated zeolite T using monoamine and triamine functional groups (M1 and M3 membranes). The M2 membrane also displayed good interfacial adhesion between impregnated Linde T and the polymer phase and successfully lies on the 2008 Robeson’s upper bound line. Overall, the present work has contributed a significant finding that is useful for the selection of an appropriate amine functionalization agent to favorably improve the interfacial adhesion and the separation properties of a membrane. In addition, the transport properties of CO_2_ and CH_4_ gases in the resultant membranes is essential in understanding the gas transport properties of CO_2_ and CH_4_ gases after the incorporation of impregnated Linde T particles using different amine functionalization agents embedded into a 6FDA-derived polymer matrix. 

## Figures and Tables

**Table 1:** Properties of fabricated pure 6FDA-derived polyimide and impregnated Linde T hybrid membranes.

Membranes	Density (g/cm^3^)	Fractional Free Volume (FFV)	Glass Transition Temperature, *T*_g_ (°C)
Pure 6FDA-derived polyimide	1.38	0.20	410.10
M1	1.29	0.24	422.80
M2	1.27	0.26	426.00
M3	1.29	0.25	425.50

**Table 2:** Gas transport properties of CO_2_ and CH_4_ gases for pure 6FDA-derived polyimide and impregnated Linde T hybrid membranes at a temperature of 30 °C and pressure of 3.5 bar.

Membranes	Permeability, *P* (barrer)		Solubility, *S* (10^−2^ cm^3^(STP)/cm^3^∙cmHg)		Diffusivity, *D* (10^−8^ cm^2^/s)		Selectivity, α		
	CO_2_	CH_4_	CO_2_	CH_4_	CO_2_	CH_4_	P_CO2_/P_CH4_	S_CO2_/S_CH4_	D_CO2_/D_CH4_
Pure 6FDA-derived polyimide	468.01	66.57	19.71	11.20	23.75	5.94	7.03	1.76	4.00
M1	765.30	56.82	21.16	7.36	36.16	7.72	13.47	2.87	4.69
M2	820.50	31.14	21.84	5.48	37.56	5.68	26.35	3.98	6.61
M3	857.70	38.12	23.90	6.22	35.88	6.13	22.50	3.84	5.86
